# Effects of Low Field Temperature on the Physicochemical Properties and Fine Structure Stability of High-Quality Rice Starch during the Grain Filling Stage

**DOI:** 10.3390/foods13193094

**Published:** 2024-09-27

**Authors:** Xutong Pang, Dongmeng Zhang, Haobo Xue, Dongping Yao, Hong Shen, Baohui Mou, Panqi Gu, Ruijuan Zhou, Fudie Meng, Jun Wu, Dongyang Lei, Bin Bai

**Affiliations:** 1 College of Agronomy, Hunan Agricultural University, Changsha 410128, China; pxt413@163.com (X.P.);; 2 State Key Laboratory of Hybrid Rice, Hunan Hybrid Rice Research Center, Changsha 410125, China; 3 College of Plant Science and Technology, Hunan Biological and Electromechanical Polytechnic, Changsha 410127, China

**Keywords:** high-quality rice, low field temperature, stability of starch quality, starch physicochemical properties and structure

## Abstract

The consumption of high-quality rice is increasing. Low temperatures during grain filling may affect the starch synthesis of high-quality rice and thus affect the quality of the rice itself. In this study, two high-quality conventional rice cultivars and two high-quality hybrid rice cultivars were selected and sown at a low temperature and normal temperature in the field. The low temperature during grain filling increased the amylose content, final viscosity, setback, short amylopectin chain ratio, and degree of amylopectin branching in four high-quality rice cultivars; meanwhile, the amylopectin content, gelatinization temperature, proportion of medium-long chain amylopectin, and the short-range order of starch decreased. Compared with the normal temperature, the alterations in the physicochemical and structural qualities of high-quality conventional rice cultivars YZX and NX42 were less significant at lower temperatures. The starch quality of high-quality conventional rice was more stable than hybrid high-quality rice.

## 1. Introduction

One of the most important food crops in the world and a staple food for Chinese people is rice (*Oryza sativa* L.) [1]. A high head rice rate, low chalkiness rate, moderate amylose content, and high sensory scores are the typical characteristics for high-quality rice. The global rice planting area is about 160 million hm^2^, and approximately 50% of China’s rice planting area is hybrid rice, which is planted on 30 million hm^2^ [2]. In the past, China’s production of high-quality rice has been relatively low, especially hybrid rice cultivars. However, in recent years, breeders have shifted from solely pursuing hybrid rice yields to emphasizing both yield and quality, and more and more high-quality hybrid rice has been promoted in the market [3].

Rice is 80% starch, which is commonly eaten by people. Starch is a general term for natural polymers consisting of amylose and amylopectin [4]. Starch’s structural and physicochemical characteristics define the quality of rice. Rice’s appearance, processing, nutritional value, and cooking qualities all affect its quality. Amylose content, chalkiness, gel consistency, and RVA profile characteristics are usually used as important indicators to evaluate rice quality. Meanwhile, starch composition, particle size, and chain length distribution also affect rice quality.

Many factors affect the quality of rice. In addition to the genetic basis of cultivars, environmental factors also play an important role. During grain filling, the average daily temperature is an important factor, with the most ideal being 23–30 °C. During grain filling, indica rice will stop filling if the temperature is lower than 20 °C [5]. During the rice grain filling stage, low temperatures increase the amylose content and affect the crystal structure of starch. At a low temperature, the amount of short-chain amylopectin increases and long-chain amylopectin is greatly reduced, which eventually decreases rice quality [6]. Low temperatures increase the proportion of small branched chains, increase the degree of branching, and reduce the average chain length of the starch, leading to poorer pasting and thermal properties, as well as reduced rice starch quality [7]. Low temperatures increase the activity of granule-bound starch synthase, which increases amylose content [8]. Low-temperature stress significantly increases the number of starch granule structures in rice, and also leads to a decrease in relative crystallinity and gelatinization enthalpy, as well as an increase in gelatinization temperature [9].

The crucial period for determining rice yield and quality is the grain filling stage, in which temperature is one of the most critical factors determining rice quality. Nowadays, some studies have made some progress in how low temperatures affect the physicochemical properties and fine structure of premium rice starch during the grain filling stage. However, there are only few studies on the stability of high-quality rice starch at a low temperature. Whether there is a difference in the stability of starch quality between high-quality conventional and hybrid rice under low-temperature stress was also the focus of this study. This study compares the physicochemical starch properties and fine structure of four high-quality rice cultivars in normal- and low-temperature sowing periods. The effects of a low temperature on the physical and chemical properties and fine structure of high-quality rice starch are studied. The stability of starch quality of different high-quality conventional and hybrid rice is evaluated after low-temperature stress, which provided a reference for the screening of low temperature resistant high-quality rice cultivars.

## 2. Materials and Methods

### 2.1. Plant Materials

Four high-quality rice cultivars were planted extensively in large areas in actual production, which were chosen for this study. These rice cultivars were Yuzhenxiang (YZX) and Nongxiang42 (NX42), three-line hybrid rice cultivar Yexiangyoulisi (YXYLS), and two-line hybrid rice cultivar Yueliangyou2646 (YLY2646). Furthermore, four rice cultivars shared a similar heading date and total growth period. The seeds used in the experiment were new for the year.

In order to subject the grain filling stage to varying temperatures, seeds were planted on three different sowing dates from April to June in 2023 at the Changsha test field of the Hunan Hybrid Rice Research Centre. Before rice transplanting, 50 kg of N-P-K compound fertilizer (N/P_2_O_5_/K_2_O = 15:15:15, N + P_2_O_5_ + K_2_O = 45%) was applied per acre. Precipitation was abundant in the year of the experiment. All the rice cultivars were planted using the conventional method in Hunan Province, with a single seedling in each clump. Each cultivar was planted in eight rows, comprising eight plants each. Water management at the seedling stage and tillering stage was carried out according to the conventional management measures of rice in Hunan Province. Dry–wet alternate irrigation was carried out at the booting stage and grain filling stage. The sowing and transplanting dates are as follows: Sowing Stage I, sown on 10 April and transplanted on 6 May; Sowing Stage Ⅱ, sown on 11 May and transplanted on 6 June; and Sowing Stage Ⅲ, sown on 12 June and transplanted on 6 July. The initial heading time of different sowing dates were about 15 July, 1 August, and 3 September.

### 2.2. Temperature Treatments and Samplings

Once the plants had commenced flowering, two automatic temperature and light HOBOs (Onset Computer Corp, Bourne, MA, USA) were positioned in the field and maintained at the centre of the plot. The HOBO recorded temperatures at 5-min intervals, and the instrument was retrieved once all material had been harvested. The field temperature data were subjected to a series of calculations in order to derive the field temperature data and to calculate the daily mean temperatures. The two sowing stages with the largest temperature gap were selected as low temperature (LT) and normal temperature (CT). The LT temperature range was 16–30 °C, and the CT temperature range was 22–34 °C. Plant materials that were harvested during the two sowing stages were used as experimental subjects in this study.

After 35 days from heading, a random selection of 100 panicles was made, then immediately dried in an oven at 38 °C until the paddy’s moisture content reached about 13%. During this process, the material was turned more often to prevent uneven drying. Subsequently, the rice grains were stored under natural conditions for a period of two months prior to undergoing subsequent experimental measurements.

### 2.3. Starch Components

Through a colourimetric method using iodine reagent, the amylose content (AC) was determined [10]. The People’s Republic of China’s national standard “GB 5009.9-2023” [11] was used to measure the total starch content. All experiments were carried out in the State Key Laboratory of Hybrid Rice.

### 2.4. Starch Isolation

Starch extraction was referenced from previously published methods [10]. Briefly, dried rice flour was soaked in 0.14% NaHSO_3_ solution for 24 h at room temperature and then ground into homogenate. The mixture was centrifuged at 5000 rpm for 15 min and the supernatant and yellowish upper sediment were discarded. An appropriate amount of MilliQ extra pure water was added and mixed with the lower white precipitate and centrifuged at 5000 rpm for 15 min. The above steps were repeated until there was no yellowish sediment. The starch was dried at 55 °C in an oven and sieved through a 200 mesh sieve and kept in a dryer. The amplitude of an indicator for the same cultivars was calculated according to the following formula: Amplitude (%) = (Value _LT_ − Value _CT_)/Value _CT_ × 100.

### 2.5. Pasting Property

Using a Rapid Visco-Analyzer (RVA) (Model RVA Super 4; Newport Scientific, Warriewood, Australia) machine, the pasting characteristics of starch were examined. With minor adjustments, the test profile was examined using the methodology outlined by previous published methods [12]. Rice starch (3 g) and 25 mL MilliQ extra pure water were mixed thoroughly in an aluminium tin and tested on the machine. The sample was first heated at 50 °C for 60 s, then heated to 95 °C for 225 s, held at 95 °C for 150 s, then cooled to 50 °C for 230 s and held at 50 °C for 85 s. Peak viscosity (PV), trough viscosity (TV), and final viscosity (FV) were the key parameters to read. Breakdown (BD = PV − TV) and setback (SB = FV − PV) were the secondary parameters [7]. Each sample was measured three times. The amplitude of an indicator for the same cultivars was calculated according to the following formula: Amplitude (%) = (Value _LT_ − Value _CT_)/Value _CT_ × 100.

### 2.6. Thermodynamic Property

Using a differential scanning calorimeter (Model Q2000; TA Instruments Ltd., Newcastle, DE, USA), the thermal characteristics of starch were determined. The precise weight of 10 mg of rice flour had to be determined in an alumina crucible. Then we added 30 µL of sterile water and seal. After that, the mixture was given a full day to acclimate to room temperature. Following this, the calorimetric scanner was employed to gradually raise the temperature by 10 °C/min from 30 °C to 95 °C. The calorimetric changes were then scanned to collate and analyse the data through the accompanying software [13]. The amplitude of an indicator for the same cultivars was calculated according to the following formula: Amplitude (%) = (Value _LT_ − Value _CT_)/Value _CT_ × 100.

### 2.7. Starch Granule Morphology and Size Analysis

Using scanning electron microscopy (SEM; Zeiss Merlin Compact, Oberkochen, Germany), the shape of starch granules was examined [14]. A malvern laser particle size analyser (Mastersizer 3000; Malvern Panalytical, Worcestershire, UK) was used to measure the starch granule size distribution [15].

### 2.8. Analysis of X-ray Diffraction (XRD)

The XRD analysis of starch was performed on an X-ray diffractometer (Rigaku Corporation, Tokyo, Japan). With a step size of 0.02° and a scanning rate of 4°/min, the starch samples were examined within the 2θ range of 4–60°. MDI Jade 5.0 was used to collect the results and calculate the relative crystallinity (%) [14].

### 2.9. Analysis of Fourier Transform Infrared Spectroscopy (FTIR)

The short-range ordered structure of rice starch was determined with the Nicolet iZ-10 Fourier Transform Infrared Spectrometer (Thermo Fisher Scientific, Inc., Waltham, MA, USA). The area between 4000 and 400 cm^−1^ was scanned 32 times for starch at a resolution of 4 cm^−1^.

### 2.10. High-Performance Anion-Exchange Chromatography (HPAEC)

The distribution of starch chain length was checked using an electrochemical detector-equipped Thermo ICS-5000 ion chromatography system (ICS5000+, Thermo Fisher Scientific, Sunnyvale, CA, USA) [10].

### 2.11. Gel Permeation Chromatography (GPC)

A Gel Chromatography-Oscillometric System was used to determine the relative molecular weight distributions of starch. We used a column heater model to keep the temperature at 80 °C. The flow rate was 0.8 mL/min [16].

### 2.12. Statistical Analysis

The mean ± standard deviation of three separate experiments was used to express the results. The obtained results were analysed by *t*-text using SPSS 25.0 statistical software to determine the significant difference at *p* < 0.05. Drawing and charting were completed using Microsoft Excel and Origin 2021.

## 3. Results and Discussion

### 3.1. Determination of Sowing Date

**Figure 1:**
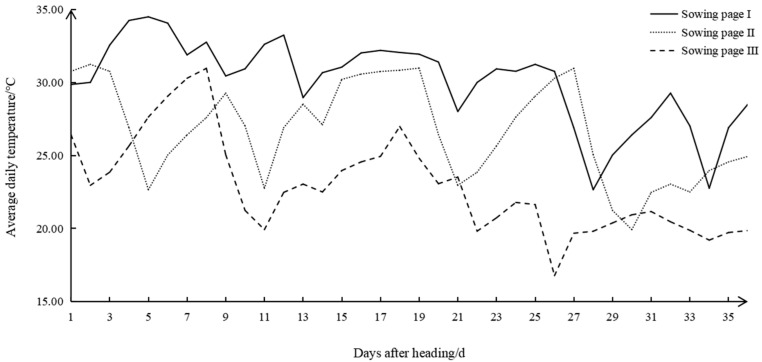
Daily mean temperature of 35 days after initial heading time for rice in three sowing stages.

The daily average temperature of 35 days after heading was counted with a HOBO recorder (Table S1). The mean daily temperature range for the 35 days following the spike initiation was 22–34 °C for Sowing Stage I, 20–31 °C for Sowing Stage II and 16–30 °C for Sowing stage Ⅲ. In Sowing Stage Ⅲ, there were 22 days with average daily temperatures below 23 °C. Thus, Sowing Stage Ⅲ was identified as low temperature (LT), and Sowing Stage I was normal temperature (CT). Therefore, this study focused on the changes in the four cultivars in Sowing Stages I and III, which had the largest temperature differences, and investigated how low field temperatures affected the physicochemical characteristics and starch structure of high-quality rice during the grain filling stage (Figure 1).

### 3.2. Amylose, Amylopectin and Total Starch Content

Compared with normal temperature, low temperature led to a considerable rise in amylose content of all cultivars, and the increase of each cultivar was different. YXYLS had the largest increase of 14.9%, while YZX had the smallest increase of 7.5%. Additionally, at a low temperature the content of amylopectin and total starch dramatically decreased during the grain filling stage. Among the four cultivars, NX42 had the largest decrease in amylopectin and total starch content, which was 13.10% and 8.17%, respectively. Meanwhile, YLY2646 had the smallest decrease, which was 6.43% and 3.19%, respectively (Table 1). Our study results were consistent with previous results showing that a low temperature in the field during the grain filling stage led to a decrease in total starch and amylopectin content [17]. The reduced activity of starch branching enzyme (SBE) and soluble starch synthase (SSS) due to low temperatures may be directly linked to the cause of the decrease in total starch and amylopectin content [18]. However, the effects of low temperature on the amylose content are controversial, with some studies reporting an increase [6] and others a decrease [7]. The effect of a low temperature on the amylose content varies with the rice cultivar itself in terms of amylose content [19].

### 3.3. Pasting Properties

Pasting properties are a crucial criterion for evaluating rice’s suitability for cooking and consumption. There is a consensus that higher PV, lower FV, and lower SB cultivars are superior for cooking and eating [20]. As shown in Table 2, low temperature had a significant effect on peak viscosity, trough viscosity, final viscosity, breakdown, and setback, but the changes were not exactly the same for different cultivars. Low temperatures in the filling stage significantly increased PV and BD of YXYLS, but significantly decreased in YLY2646, while the other two cultivars did not change significantly. At the same time, low temperatures led to a significant increase in TV of YXYLS and no significant change in TV of other cultivars. A low temperature during the filling stage caused the FV of all four cultivars to rise greatly. The changes in FV may be closely related to changes in the crystalline structure of starch [21]. Each cultivar’s SB increased significantly at a low temperature, indicating that temperature has a big impact on SB. Low temperature can promote starch retrogradation and accelerate starch ageing, leading to the increase in the SB [7]. In this study, the pasting properties and cooking quality of the four cultivars became worse under low temperature conditions at the filling stage. Among them, YXYLS and YLY2646 had large and significant variation and poor stability, while YZX and NX42 had small variation and the better stability.

### 3.4. Thermal Properties

There is a strong relationship between the cooking and eating characteristics of rice starch and its thermal properties. The difficulty of cooking refined rice is reflected in its gelatinization temperature. The higher the gelatinization temperature is, the more heat it needs to absorb during the cooking process of rice [10,15]. In this study, it was found that the gelatinization enthalpy (∆H), onset (To), peak (Tp), and final (Tc) of rice starch in four high-quality rice starches were reduced at a low temperature, but the significance of the reduction was different. They decreased significantly in all four cultivars at a low temperature, and Tp and Tc decreased significantly in the other three cultivars except YZX. Only YZX showed a significant decrease in ∆H, while the other three cultivars showed no significant change at low temperature (Table 3). In the previous research, a low temperature during grain filling lead to a decrease in the enthalpy and temperature of pasting for all cultivars [6,22]. Also, variations in pasting temperature and enthalpy were different for different high-quality rice cultivars. Therefore, YLY2646 had the largest amplitude up to 13.96%, while YZX had the smallest amplitude. In general, the thermal stability of YLY2646 is worse, and YZX is better.

### 3.5. Starch Granule Morphology and Particle Size Distribution

**Figure 2:**
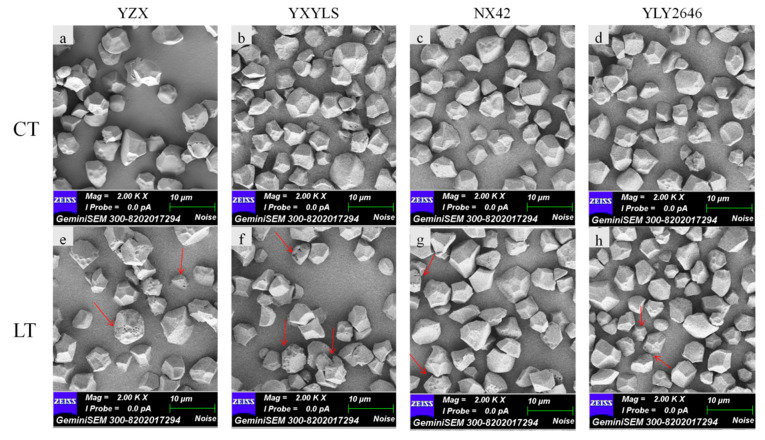
Effects of low temperature during grain filling on starch scanning electron photomicrographs. Note: (**a**,**e**) indicate CT, LT of YZX, respectively; (**b**,**f**) indicate CT, LT of YXYLS, respectively; (**c**,**g**) indicate CT, LT of NX42, respectively; (**d**,**h**) indicate CT, LT of YLY2646, respectively. The red arrows mean microporous starch granules. Scale bar = 10 μm.

The starch granule morphologies of all cultivars were similar. According to the results of scanning electron microscopy (SEM), they are all irregular polyhedrons (Figure 2), although there were some differences in the surface of the granules between normal temperature and low temperature. The starch granules at the normal temperature were irregular, angular, and smooth, whereas the low temperature starch granules’ surface featured visible micropores. The unevenness of the entire granule surface could be attributed to the low temperature’s slow development of amyloplasts [7]. Plant cultivars and climatic conditions can cause variations in the shape of starch granules [23]. The morphological development of starch granules can affect rice quality [24].

The surface area, number, and volume distribution of starch granules were classified into two categories: small and medium starch granules (d < 10 μm) and large starch granules (d ≥ 10 μm) [25]. The surface area, quantity, and volume distribution of the four high-quality rice cultivars showed a typical single-peak curve peak at 6–13 μm, and the curve coincidence degree of NX42 under CT and LT conditions was higher, indicating that a low temperature had little effect on the starch particle size distribution of NX42, and the stability of this variety was better (Figure S1). LT significantly increased the percentage of small granule starch in YXYLS, NX42 and YLY2646, while decreased the percentage of small granule starch in YZX (Table 4), which may be caused by the decrease of amyloplast and the loosening of starch granules caused by the low temperature [26]. A prior study revealed that low temperature treatment during the filling stage increased the proportion of small and medium starch granules, decreased the proportion of large granules, and decreased the average diameter of starch granules [6]. The amplitude of starch particle size distribution variation varies between cultivars. Compared with CT, under LT treatment, the largest variation of starch granule volume percentage, surface area percentage, and number percentage was YLY2646, and the smallest was NX42 (Table 4). Therefore, it can be considered that the starch particle size distribution of NX42 was the most stable under LT conditions, which is consistent with the above conclusions.

### 3.6. Crystal Structure

**Figure 3:**
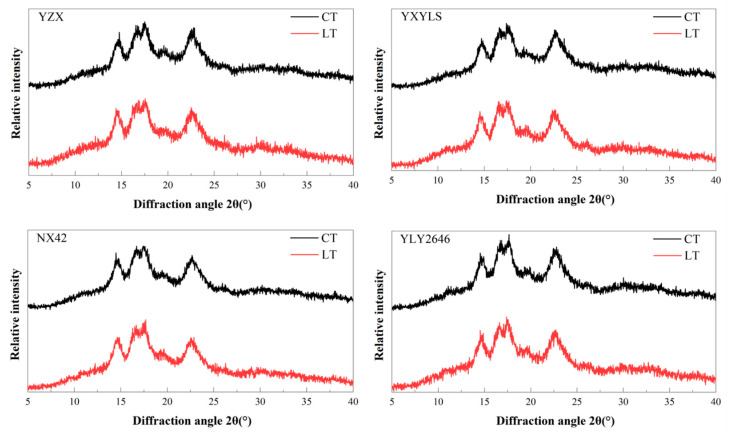
Effects of low temperature on the X-ray diffraction pattern of rice starch.

An X-ray diffraction technique was employed to examine the properties of rice starch crystals [27]. None of the cultivars changed under CT and LT during the grain filling stage (Figure 3). There was a single peak at 15°2θ and 23°2θ, respectively, while the twin peaks at 17°2θ and 18°2θ corresponded to starch crystals of the A-type. This kind of rice starch crystal was unaffected by the low temperature during the grain filling stage. Through analysis of starch crystallinity, it was found that the relative crystallinity of YLY2646 decreased slightly but not significantly under LT treatment, while the relative crystallinity of the other three cultivars increased. In this study, compared with CT, the relative crystallinity of the four types of starch under LT conditions did not change significantly, which may be connected to the degree of polymerization, amylose content, amylopectin chain length distribution, and field temperature [9].

The amorphous portion of the starch granules is associated with the intensity at 1022 cm^−1^ of the FTIR spectrum, whereas the crystalline portion of the granules is associated with the intensity at 1045 cm^−1^. A useful ratio to determine the short-range ordered structure of starch is 1045/1022 cm^−1^; the higher the ratio, the greater the short-range ordering in the starch [28]. LT caused a significant decrease in 1045/1022 cm^−1^ in all four cultivars, and the degree of starch short-range ordering decreased (Table 5). Among them, YLY2646 had the largest change of 6.94% and NX42 had the smallest change of 4.23%. In conclusion, YLY2646 underwent the most alteration in starch internal structure upon LT treatment, whereas NX42 underwent the least alteration and exhibited the highest degree of stability.

### 3.7. Chain Length Distribution of Amylopectin

**Figure 4:**
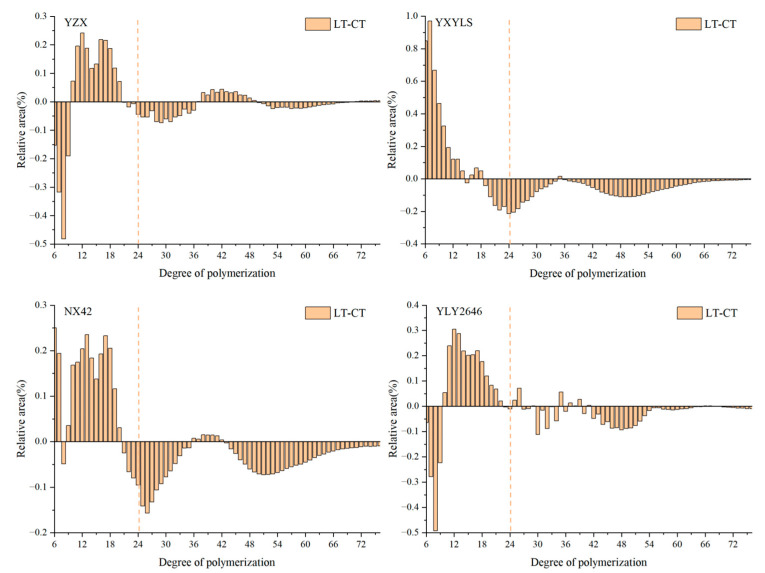
Effect of low temperatures during grain filling on the relative amylopectin chain distributions.

Based on the degree of polymerization, amylopectin is typically classified into four types: A chain (6 ≤ DP ≤ 12), B_1_ chain (13 ≤ DP < 24), B_2_ chain (24 ≤ DP < 37), and B_3_ chain (DP ≥ 37). A and B_1_ chain are short chain, while B_2_ and B_3_ chain are long chain. Compared with CT, the change trend of long and short chain amylopectin distribution of the four high-quality rice cultivars under LT treatment was basically the same. The proportion of short-chain amylopectin (6 ≤ DP < 24) increased in all four high-quality rice cultivars under LT treatment, while the proportion of long-chain (24 ≤ DP ≤ 37) amylopectin decreased in all of them (Figure 4). Amylopectin is an important component of rice starch, and the ratio of long to short amylopectin chains has a major effect on rice quality [16]. During the grain filling stage, the low temperature decreased the amount of total starch, increased the amount of short chain (DP6-13) in the components of amylopectin, and also decreased the amount of medium chain (DP20-27) and long chain (DP44-54) [29]. The ratio of amylopectin containing long chains was high; starch granules could not be fully gelatinized, while the highest viscosity and disintegration value would be lower, which affected the taste of the rice [30]. The ratio of amylopectin containing short chain parts is high, which is conducive to starch gelatinization, making it easy to form a higher maximum viscosity and disintegration value, which is conducive to the improvement of rice taste. Interestingly, the four cultivars in this study showed a decrease in long chain and an increase in short chain amylopectin content following LT treatment, which somewhat enhanced the rice’s pasting qualities. However, the distribution of amylopectin chain length did not significantly change. Appropriately low temperatures in the field during the grain filling stage may be beneficial for improving rice pasting characteristics.

### 3.8. Relative Molecular Weight Distribution of Starch

**Figure 5:**
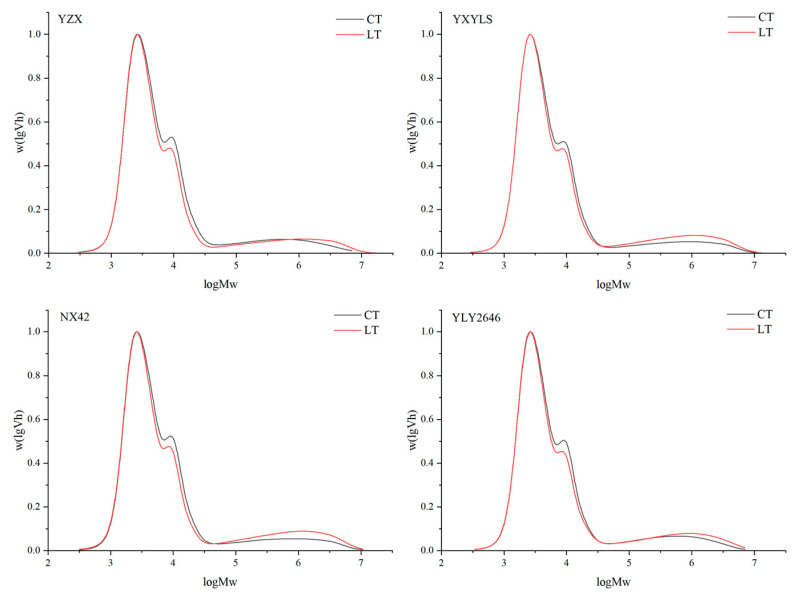
Effect of low temperatures during grain filling on the relative molecular weight distribution of amylopectin chains.

A method for determining the relative molecular weight distribution of rice starch is gel permeation chromatography (GPC). According to GPC, the first peak Ap_1_ (DP < 30) is composed of short chains of amylopectin, the second peak Ap_2_ (30 ≤ DP < 100) represents a longer amylopectin branch chain, and the third small peak AM (100 ≤ DP < 20,000) is an amylose chain [31]. It demonstrates that a low temperature at the filling stage reduced the AP_2_ peak of the four cultivars, and the AM peak increased, suggesting that the rice’s long chain of amylopectin was less present due to the low temperature during the filling stage, while the content of amylose increased, which was consistent with the amylose content measured in this study (Figure 5). The degree of amylopectin branching is indicated by the ratio of Ap_1_ to Ap_2_. The higher the ratio, the more branches, while the more branches of straight chain amylopectin, the better the flavour of the rice [32]. Under LT treatment, the AP_1_/AP_2_ of the four high-quality rice cultivars increased, and the branching degree of amylopectin became higher (Table 6). Appropriate low temperatures in the field during the grain filling stage may have a positive effect on rice quality. NX42 showed the greatest increase in amylopectin branching degree; its quality remained stable, and it was well adapted to low temperatures in the field throughout the grain filling period.

## 4. Conclusions

This study elucidated the effects of low field temperature on the physicochemical properties and fine structure stability of high-quality rice starch during the grain filling stage. Low temperature during the filling period negatively affected the appearance and structure of high-quality rice, producing micropores on the surface of starch granules and increasing the proportion of small-grained starch. The rice starch crystal type did not change between LT and CT. At low temperatures, the pasting properties of all cultivars deteriorated, with higher final viscosity and setback. Low filling temperatures led to higher amylose content, lower gelatinization temperatures, and enthalpy. A higher proportion of short chains and lower proportion of long chains in amylopectin improved the rice’s pasting qualities under low temperatures during the filling stage. Meanwhile, the higher branching degree of amylopectin could also beneficial of the rice’s quality. In addition, the starch physicochemical properties of high-quality conventional rice cultivars (YZX and NX42) were relatively stable under low temperatures in the field during the grain filling stage, with smaller changes in indicators, while the starch quality stability of the high-quality hybrid rice cultivars (YXYLS and YLY2646) was poor, which may be related to the difference in parental genotype of hybrid rice. Overall, this study has provided an evidence to enhance the quality of high-quality rice and to target the improvement of hybrid rice quality.

## Figures and Tables

**Table 1:** Effects of a low temperature during grain filling on starch composition.

Cultivar	Treatment	Amylose Content (%)	Amylopectin Content (%)	Total Starch Content (%)
YZX	CT	12.90 ± 0.26 b	62.82 ± 0.65 a	75.72 ± 0.40 a
	LT	13.87 ± 0.29 a	57.99 ± 1.90 b	71.86 ± 1.84 a
YXYLS	CT	11.90 ± 0.26 b	65.77 ± 1.97 a	77.67 ± 2.07 a
	LT	13.67 ± 0.29 a	58.41 ± 1.05 b	72.08 ± 1.31 a
NX 42	CT	15.33 ± 0.15 b	61.89 ± 1.30 a	77.22 ± 1.27 a
	LT	17.13 ± 0.15 a	53.78 ± 0.80 b	70.91 ± 0.93 b
YLY 2646	CT	14.53 ± 0.12 b	56.58 ± 0.57 a	71.11 ± 0.62 a
	LT	15.90 ± 0.00 a	52.94 ± 0.76 b	68.84 ± 0.76 a

Data are shown as the mean ± standard deviation of three replicates. Different letters for the same cultivar in the same column mean significantly different (*p* < 0.05). CT, normal temperature; LT, low temperature.

**Table 2:** Effects of a low temperature during grain filling on pasting property.

Cultivar	Treatment	PV (cP)	TV (cP)	BD (cP)	FV (cP)	SB (cP)
YZX	CT	2152 ± 57 a	1377 ± 39 a	774 ± 95 a	2064 ± 33 b	687 ± 6 b
	LT	2274 ± 16 a	1364 ± 13 a	910 ± 30 a	2313 ± 12 a	949 ± 1 a
YXYLS	CT	901 ± 33 b	617 ± 27 b	284 ± 6 b	1080 ± 37 b	463 ± 10 b
	LT	2237 ± 14 a	1440 ± 31 a	797 ± 46 a	2396 ± 19 a	955 ± 16 a
NX42	CT	2472 ± 18 a	1735 ± 49 a	738 ± 32 a	2567 ± 44 b	832 ± 6 b
	LT	2498 ± 31 a	1743 ± 8 a	755 ± 32 a	2763 ± 11 a	1020 ± 8 a
YLY2646	CT	2712 ± 21 a	1712 ± 35 a	1001 ± 41 a	2613 ± 31 b	902 ± 7 b
	LT	2363 ± 25 b	1631 ± 35 a	733 ± 18 b	2776 ± 34 a	1146 ± 6 a

Data are shown as the mean ± standard deviation of three replicates. Different letters for the same cultivar in the same column mean significantly different (*p* < 0.05). CT, normal temperature; LT, low temperature.

**Table 3:** Effects of a low temperature during grain filling on thermal property.

Cultivar	Treatment	T_o_ (°C)	T_P_ (°C)	T_c_ (°C)	ΔH (J/g)
YZX	CT	71.86 ± 0.22 a	77.13 ± 0.34 a	81.68 ± 0.55 a	6.16 ± 0.16 a
	LT	69.84 ± 0.56 b	75.48 ± 0.44 a	79.79 ± 0.42 a	5.35 ± 0.22 b
YXYLS	CT	71.54 ± 0.65 a	77.61 ± 0.53 a	82.20 ± 0.59 a	5.43 ± 0.32 a
	LT	69.31 ± 0.08 b	75.39 ± 0.16 b	80.15 ± 0.20 b	5.43 ± 0.32 a
NX42	CT	71.04 ± 0.13 a	76.41 ± 0.12 a	81.20 ± 0.15 a	5.92 ± 0.03 a
	LT	67.57 ± 0.14 b	73.07 ± 0.16 b	77.87 ± 0.21 b	5.81 ± 0.20 a
YLY2646	CT	71.41 ± 0.26 a	77.19 ± 0.19 a	81.23 ± 0.95 a	5.23 ± 0.74 a
	LT	65.44 ± 0.14 b	72.54 ± 0.12 b	77.41 ± 0.10 b	4.50 ± 0.27 a

Data are shown as the mean ± standard deviation of three replicates. Different letters for the same cultivar in the same column mean significantly different (*p* < 0.05). CT, normal temperature; LT, low temperature.

**Table 4:** Effects of low temperature during grain filling on starch granule size distribution.

Cultivar	Treatment	Volume Percentage/%		Surface Area Percentage/%		Number Percentage/%	
		d < 10 μm	d ≥ 10 μm	d < 10 μm	d ≥ 10 μm	d < 10 μm	d ≥ 10 μm
YZX	CT	29.71 ± 0.76 a	70.29 ± 0.76 b	58.17 ± 0.04 a	41.83 ± 0.04 b	93.24 ± 0.04 a	6.76 ± 0.04 b
	LT	24.34 ± 0.21 b	75.66 ± 0.21 a	48.13 ± 0.02 b	51.87 ± 0.02 a	89.88 ± 0.15 b	10.12 ± 0.15 a
YXYLS	CT	26.25 ± 0.12 b	73.75 ± 0.12 a	55.92 ± 0.42 b	44.08 ± 0.42 a	94.04 ± 0.01 b	5.96 ± 0.01 a
	LT	33.62 ± 0.31 a	66.38 ± 0.31 b	65.48 ± 0.03 a	34.52 ± 0.03 b	96.58 ± 0.05 a	3.42 ± 0.05 b
NX42	CT	27.84 ± 0.05 b	72.16 ± 0.05 a	57.30 ± 0.22 b	42.70 ± 0.22 a	93.67 ± 0.06 a	6.33 ± 0.06 a
	LT	30.61 ± 0.28 a	69.39 ± 0.28 b	58.89 ± 0.10 a	41.11 ± 0.10 b	93.83 ± 0.03 a	6.17 ± 0.03 a
YLY2646	CT	28.49 ± 0.44 b	71.51 ± 0.44 a	56.03 ± 0.24 b	43.97 ± 0.24 a	92.63 ± 0.24 b	7.37 ± 0.24 a
	LT	37.41 ± 0.51 a	63.59 ± 0.51 b	66.04 ± 0.23 a	33.96 ± 0.23 b	95.51 ± 0.05 a	4.49 ± 0.05 a

Data are shown as the mean ± standard deviation of three replicates. Different letters for the same cultivar in the same column mean significantly different (*p* < 0.05). CT, normal temperature; LT, low temperature.

**Table 5:** Effects of low temperature during grain filling on crystal structure.

Cultivar	Treatment	Degree of Crystallinity/%	Crystal Pattern	1045/1022 cm^−1^
YZX	CT	19.14 ± 0.15 a	A	0.69 ± 0.003 a
	LT	19.40 ± 0.12 a	A	0.65 ± 0.003 b
YXYLS	CT	17.11 ± 0.17 a	A	0.67 ± 0.013 a
	LT	17.43 ± 0.14 a	A	0.63 ± 0.003 b
NX42	CT	20.55 ± 0.08 a	A	0.71 ± 0.007 a
	LT	21.83 ± 0.12 a	A	0.68 ± 0.008 b
YLY2646	CT	16.64 ± 0.13 a	A	0.72 ± 0.004 a
	LT	16.57 ± 0.11 a	A	0.67 ± 0.004 b

Data are shown as the mean ± standard deviation of three replicates. Different letters for the same cultivar in the same column mean significantly different (*p* < 0.05). CT, normal temperature; LT, low temperature.

**Table 6:** Effects of low temperatures during grain filling on relative molecular weight distributions.

Cultivar	Treatment	Ap_1_ (%)	Ap_2_ (%)	AM (%)	Ap_1_/Ap_2_
YZX	CT	67.46 ± 0.34 a	21.74 ± 0.22 a	10.79 ± 0.24 b	3.10 ± 0.03 b
	LT	67.08 ± 0.28 a	19.60 ± 0.17 b	13.32 ± 0.11 a	3.42 ± 0.16 a
YXYLS	CT	67.62 ± 0.22 a	22.16 ± 0.39 a	10.22 ± 0.52 b	3.05 ± 0.13 b
	LT	65.57 ± 0.35 a	19.13 ± 0.16 b	15.31 ± 0.22 a	3.43 ± 0.14 a
NX42	CT	66.58 ± 0.21 a	22.69 ± 0.41 a	10.74 ± 0.29 b	2.93 ± 0.11 b
	LT	64.86 ± 0.19 a	18.67 ± 0.35 b	16.47 ± 0.27 a	3.47 ± 0.06 a
YLY2646	CT	66.57 ± 0.15 a	21.57 ± 0.27 a	11.86 ± 0.17 b	3.09 ± 0.08 b
	LT	66.53 ± 0.24 a	19.65 ± 0.32 b	13.83 ± 0.17 a	3.39 ± 0.02 a

Data are shown as the mean ± standard deviation of three replicates. Different letters for the same cultivar in the same column mean significantly different (*p* < 0.05). CT, normal temperature; LT, low temperature.

## Data Availability

The original contributions presented in the study are included in the article/Supplementary Materials, further inquiries can be directed to the corresponding authors.

## Supplementary Materials

The following supporting information can be downloaded at: https://www.mdpi.com/article/10.3390/foods13193094/s1, Figure S1: Effects of low temperature on the surface area distribution, number distribution and volume distribution of rice starch granules; Table S1: Daily mean temperature of 35 days after initial heading time for rice in three sowing stages.
