# Supplementary material for: Effects of Low Field Temperature on the Physicochemical Properties and Fine Structure Stability of High-Quality Rice Starch during the Grain Filling Stage
Source: Foods. 2024 Sep 27;13(19):3094. doi: 10.3390/foods13193094 (PMC11475225; doi:10.3390/foods13193094)
Supplement: Supplementary file 1 [file foods-13-03094-s001.zip › Supplementary Figure S1.pdf]

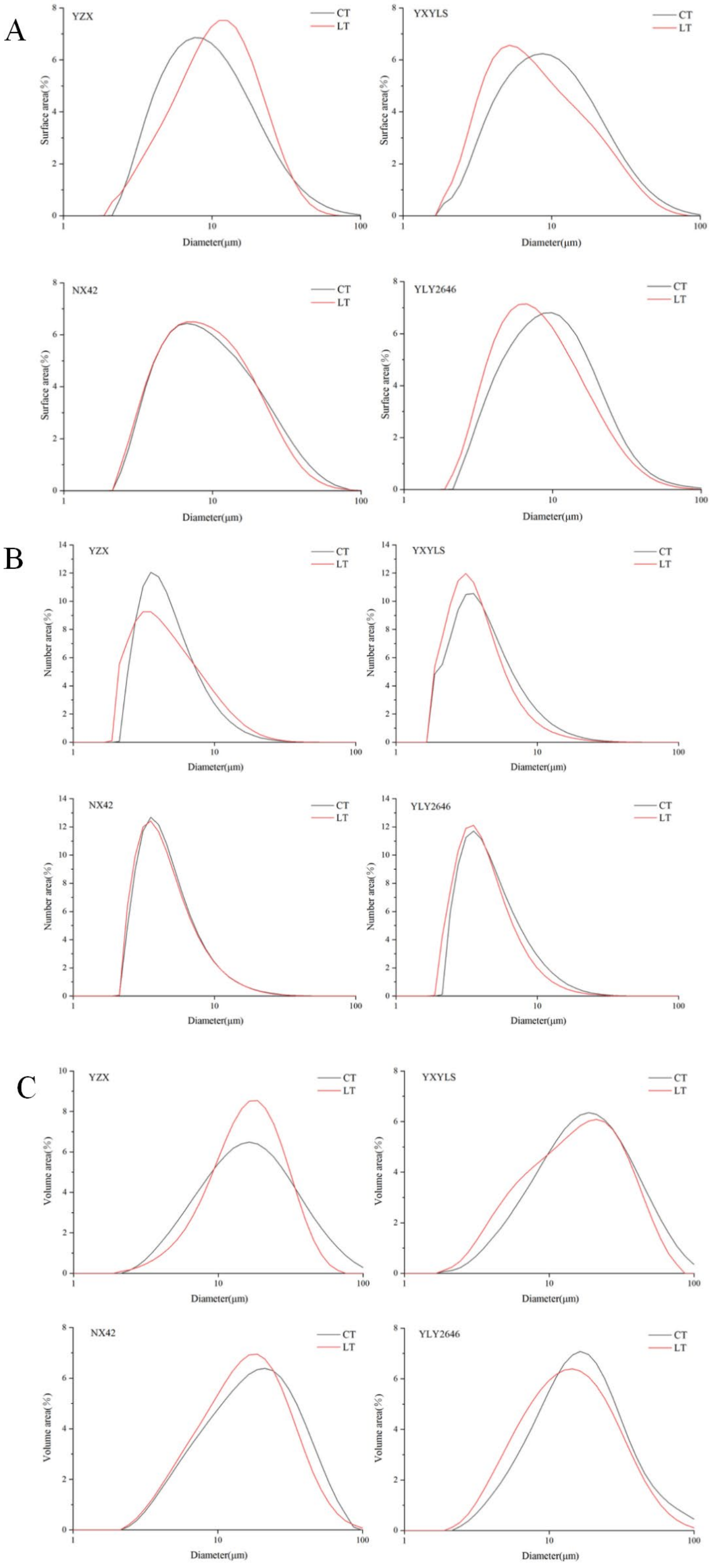

Figure S1. Effects of low temperature on the surface area distribution, number distribution and volume distribution of rice starch granules. Note: (A) represent the starch granule surface area distributions for all samples; (B) represent the starch granule number distributions for all samples; (C) represent the starch granule volume distributions for all samples.
